# A Novel Method for Preparing Uniform Micro-Sized Dry Powder Formulations, Including Aggregation-Controlled VHH

**DOI:** 10.3390/antib14020029

**Published:** 2025-03-31

**Authors:** Tatsuru Moritani, Hidekazu Masaki, Ryo Yonehara, Takeru Suzuki, Hidenao Arai, Masayuki Tsuchiya, Naoto Nemoto

**Affiliations:** 1 RICOH Company, Ltd., 2-7-1 Izumi, Ebina, Kanagawa 243-0460, Japan; 2 Epsilon Molecular Engineering Inc., 255 Shimo-okubo, Sakura-ku, Saitama 338-8570, Japan; masaki@epsilon-mol.co.jp (H.M.); yonehara@epsilon-mol.co.jp (R.Y.); arai_h@epsilon-mol.co.jp (H.A.);

**Keywords:** nanobody, single-domain antibody, fine droplet drying process, microparticle, inhalable powder, intratracheal administration, inkjet technology

## Abstract

Background: The preparation of antibodies in powder form without changing their physicochemical properties may enable their use in new drug delivery system therapies or non-refrigerated storage. The variable domain of heavy-chain antibodies (VHHs) is more suited for this purpose than that of conventional antibodies because of VHHs’ high thermal stability and ability to refold. Methods: In this report, the fine droplet drying (FDD) process was selected as the powderization technique because of its favorable features, such as mild drying conditions and the generation of uniform particle sizes. The aggregation, binding, particle, and in vitro inhalation properties of the prepared VHH powders (VHHps) were evaluated. Results: The amount of aggregated VHHs present in the VHHps depended on the flow temperature during the FDD process, with higher temperatures yielding a higher aggregation ratio. In contrast, no significant difference in binding activity was observed between each VHHp preparation and the native VHHs. However, this process degraded VHHs or inactivated their function, and ultimately, only about 30% of the original VHHs were functional, whereas the remaining VHHs that were not degraded showed little loss of functionality, even after storage at room temperature for more than two years. Analysis of the VHHp samples revealed that the particles were uniformly spherical with a single-micron size. The VHHps showed fine inhalation properties in the inhalation property test. Conclusions: These findings suggest that the FDD process affords various VHH powder formulations, including pharmaceutical formulations.

## 1. Introduction

Heavy chain-only antibodies (HCAbs) were initially discovered in the blood of camelids by Hamers et al. in 1993 [1], revealing that 20–50% of camelid antibodies are HCAbs, comprising a dimer of an identical heavy-chain polypeptide and no light chains [2,3,4,5,6]. The variable domain of HCAbs (VHHs) and its recombinant expression, has attracted attention. VHHs have superior thermal stability and refolding ability due to their lower molecular weight compared to conventional antibodies, although recent reports have shown that they do not always have thermal stability or a refolding ability [7]. Moreover, VHHs are a major attraction because of the ease of molecular design through protein engineering. By 2024, 4 VHH-based pharmaceuticals have been approved: Cablivi (Caplacizumab, 2019), Carvikti (CAR-T, 2022), Nanozora (Ozoralizumab, 2022) and Envida (Envafolimab, 2021), more than 20 nanobody-related drugs have entered clinical stages (https://www.sinobiological.com/resource/antibody-technical/nanobody, accessed on 26 November 2024).

Already in 1995 a first camelized human VH synthetic library was constructed (Riechmann and Winter group) [8]. Furthermore, in recent years, from the perspective of animal welfare, there has been a growing trend toward producing antibodies without us-ing animals [9]. Therefore, we constructed a simple synthetic VHH library to obtain VHHs in a test tube without immunizing camelids, and conducted selection experiments via evolutionary molecular engineering that resulted in the selection of anti-survivin VHHs [10]. Several synthetic VHH libraries have recently been designed, and the in vitro selection of VHHs without immunizing animals has been successful [11]. We recently constructed a novel humanized synthetic VHH library (Pharma Logical^®^ Library, Epsilon Molecular Engineering, Saitama, Japan) that allows us to obtain VHHs with high affinity and specificity against various antigens, including membrane proteins for drug discovery [12].

The COVID-19 pandemic increased concern about the inhalation delivery technique to the lungs. In particular, DPI (dry powder inhaler) is a promising dosage form because it can be more economical, environmentally friendly, and easier for patients to use themselves compared with other delivery techniques. However, DPI generally requires a precise particle size of an inhalable powder for efficient delivery to the lungs, i.e., 1–5 mm diameter [13]. In general, inhalation drugs often have a wide distribution of particle sizes, resulting in variations in the amount of drug reaching the lungs, and this issue affects their therapeutic effect. Furthermore, unintended larger particle sizes can cause inertial impaction and deposition in the oral cavity, pharynx, or larynx, increasing the frequency of side effects [14]. Therefore, uniformly controlled powder formulation is desired to resolve this issue.

The FDD (fine droplet drying) process, a unique powderization technique using inkjet technology, was developed for designing functional micron-sized particles [15]. Several studies of the FDD process applied to pharmaceutical formulation have been reported [16]. A feature of this process is the size uniformity of discharged droplets from the inkjet head. The diameter of the discharged droplets is quite fine, approximately 10 mm, and considerably uniform. Such a feature should facilitate milder air drying than general spray drying methods. In a previous study, the FDD process was applied to develop a salmon calcitonin-loaded inhalable powder [17]. The salmon calcitonin included in the powder was found to have no significant conformational changes and did not aggregate. Thus, the FDD process can potentially be used to develop powder formulations with biopharmaceuticals.

In this context, combining VHHs with the FDD process can potentially deliver VHH particles deep into the lungs, making delivery effective against respiratory diseases. For example, VHHs that neutralize viruses such as severe acute respiratory syndrome coronavirus 2, respiratory syncytial virus and influenza, bacteria that cause nontuberculous mycobacteria lung disease, and fungi causing pulmonary mycosis can be utilized as therapeutic agents to treat respiratory infections.

In this report, the applicability of the FDD process for preparing inhalable dry powder formulations of a VHH was investigated. The FDD process successfully produced VHH-loaded powders (VHHps) under mild drying conditions, achieving powderization without adverse effects such as aggregation and reduced binding activity. Several VHHps were evaluated based on their aggregation, binding, particle, and in vitro inhalable properties.

## 2. Materials and Methods

### 2.1. Chemicals

D-mannitol for molecular biology was purchased from Molecular Depot LLC (San Diego, CA, USA). The VHH against human serum albumin (HSA) (anti-HSA-VHH) used in this study was screened from a synthetic library (Pharma Logical^®^ Library, Epsilon Molecular Engineering, Saitama, Japan) by Epsilon Molecular Engineering Inc. and produced by Ajinomoto Co., Inc. (Tokyo, Japan). Distilled water, human serum albumin, phosphate-buffered saline (PBS, pH 7.4), and Tween-20 were obtained from Fujifilm Wako Pure Chemical Corporation (Osaka, Japan).

### 2.2. Preparation of the VHH-Loaded Powders

Powderization was performed using the previously reported FDD procedure [15]. Briefly, mannitol (6.5 mg) was dissolved in a solution of 0.1× PBS with the VHH (1300 mg), with a total solid content of 2% (*w*/*w*). The solution was magnetically stirred for 1 h on a ceramic hot stirrer (AS ONE Corp., Osaka, Japan) and filtered through a polytetrafluoroethylene filter with a 1 µm pore size (Millipore Corporation, Burlington, MA, USA). Droplets of sample solution were generated using a customized inkjet head based on MH2420 (RICOH, Tokyo, Japan) and dried with hot air under the following conditions: driving frequency and voltage of piezo element at 310 kHz and 10 V, respectively, and an airflow rate of 50 m^3^/h. Three air temperature conditions (i.e., 35, 50, and 65 °C) were chosen for test aggregation of the VHH during powderization using the FDD process. The three VHHp samples were prepared using the same method and conditions, except for the airflow temperature. The VHHps can be easily redissolved using standard phosphate buffer or Good’s buffers.

### 2.3. Physicochemical Evaluation

#### 2.3.1. High-Performance Liquid Chromatography (HPLC) Analysis

The aggregation rate of the anti-HSA-VHH in the VHHps was analyzed using size exclusion chromatography (SEC) with an ACQUITY UPLC H-Class PLUS Bio System (Waters, Milford, MA, USA). The analysis was performed using an ACQUITY Premier Protein SEC column (250 Å, 1.7 μm, 4.6 × 150 mm, Waters) according to the manufacturer’s instructions.

#### 2.3.2. Bio-Layer Interferometry

The binding affinity between the anti-HSA-VHH and HSA was measured using an Octet RED 384 instrument (Sartorius, Göttingen, Germany) and analyzed using Octet software, v1.2.1.5 (Molecular Devices, San Jose, CA, USA). The anti-HSA-VHH in PBS-T (PBS plus 0.05% Tween-20, pH 7.4) was immobilized on an Anti-Penta-HIS (HIS1K) biosensor chip according to the manufacturer’s instructions. Various concentrations (200, 100, 50, 25, 12.5, 6.25, and 3.13 nmol/L) of analyte solutions containing HSA were loaded.

#### 2.3.3. Scanning Electron Microscopy (SEM)

The surface morphology of the VHHps was evaluated using a VE-8800 instrument (KEYENCE Corp., Osaka, Japan). Platinum/palladium coating was carried out using an E-1030 magnetron sputtering device (Hitachi Ltd., Tokyo, Japan). The thickness of the platinum/palladium coating was 10 nm, and the accelerating voltage was 5 kV in the SEM experiment.

#### 2.3.4. Laser Diffraction

A Microtrac MT3000II (MicrotracBel, Osaka, Japan) particle size measuring system with laser diffraction under dry conditions was used to measure the particle sizes of the VHHps at a pressure of 0.2 MPa. The span factor was calculated as SPAN = (*d*_90_ − *d*_10_)/*d*_50_, where *d*_10_, *d*_50_, and *d*_90_ represent the diameter of the particle size at 10%, 50%, and 90%, respectively, which is the calculated volume.

#### 2.3.5. Andersen Cascade Impactor

Cascade impactor analysis was carried out to estimate the inhalation properties of the VHHps using an AN-200 system (Tokyo Dylec Corp., Tokyo, Japan), as shown in Figure S1. Thirty milligrams of sample filled a JP No. 2 hard capsule of hydroxypropyl methylcellulose, and the capsule was installed in a JetHaler^®^ (Tokico System Solutions, Ltd., Kanagawa, Japan). The formulation in each capsule was dispersed through the device at 28.3 L/min for 10 s × 3 times, and the amount of sample in each stage (stages 0–7) and that in the capsule were measured using an electronic balance (Shimadzu Corporation, Tokyo, Japan). The FPF (fine particle fraction) value was defined as the ratio of total drug deposited in stage 2 and lower.

## 3. Results and Discussion

### 3.1. Appearance and Particle Size Distribution of VHHps

**Figure 1:**
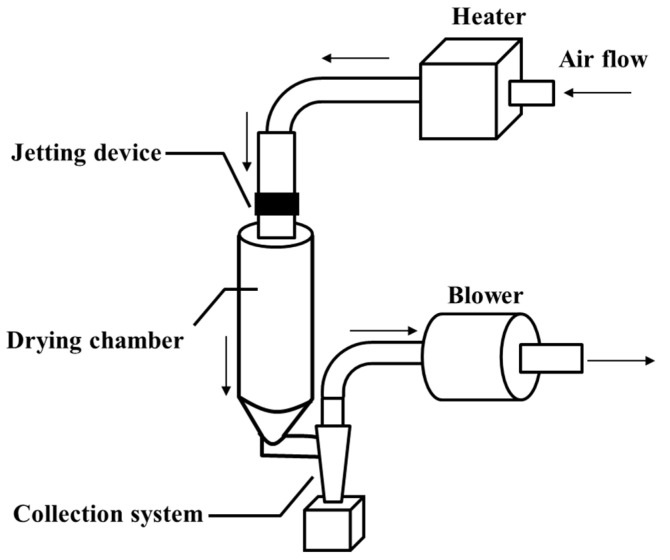
Schematic diagram of the FDD process. The arrows indicate the air flow.

**Figure 2:**
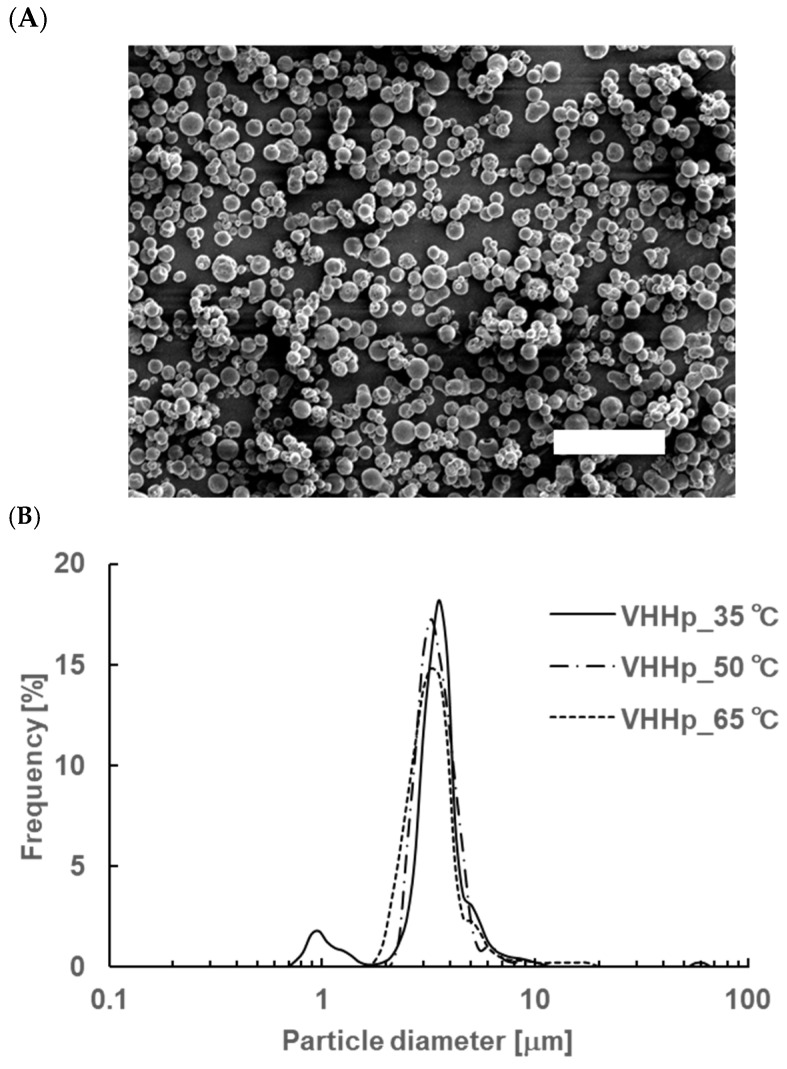
The observation of a VHHp and its particle size distribution. (**A**) SEM image of a VHHp prepared under drying conditions of 35 °C. The white scale bar represents 20 ηm. (**B**) Particle size distribution of a VHHp via laser diffraction analysis. Each VHHp was prepared under drying conditions of 35 °C, 50 °C, and 65 °C.

The expected drawbacks (e.g., aggregation and degradation) of VHHs through the powderization process were the main concerns in this study [18]. Thus, mild air-drying conditions were preferred to avoid such drawbacks. The schematic diagram of the FDD process adapted for the powderization of the VHHs is shown in Figure 1. VHHps (anti-HSA-VHH) were produced through the FDD process with air temperatures of 35, 50, and 65 °C, which are relatively low compared with the general temperatures used in the spray-drying process [19]. SEM analysis revealed that each VHHp was a spherical particle with a uniform size (Figure 2A). From the results of laser diffraction analysis, the particle diameter of the VHHps prepared under air drying conditions of 35, 50, and 65 °C were 3.6, 3.4, and 3.4 mm, and the SPAN factor values of the VHHps were calculated to be 0.7, 0.5, and 0.7, respectively (Figure 2B). In a previous study, the relationship between mannitol particle morphology and airflow temperature was investigated [20], revealing that the particle morphology was influenced by the airflow temperature, with a high temperature of 120 °C generating a rough surface. In contrast, the VHHp prepared with an airflow of 35 °C adopted a spherical shape and smooth surface. This difference in the drying conditions may be responsible for the observed size uniformity of the discharged droplets. Higher drying temperatures generally generate greater polydispersity, with larger droplets formed compared with the average droplet size. In contrast, monodispersed droplets, like those prepared via the FDD process, may not require extra heat during drying. Previous studies have produced and evaluated several kinds of pharmaceutical powders using the FDD process [15,17], with mild airflow conditions as a standard approach. In this context, the FDD process may effectively control the surface morphology of VHHps, possibly leading to the fine flowability of the powder because of its small contact area [21]. Moreover, the powderization of VHHs may improve their storage stability and yield uniform solubility derived from particle size uniformity [22,23]. Thus, the FDD process represents a potential option for preparing drug-loaded fine powder formulations, especially heat-sensitive drugs such as biomolecules.

### 3.2. VHH Properties in VHHps

**Figure 3:**
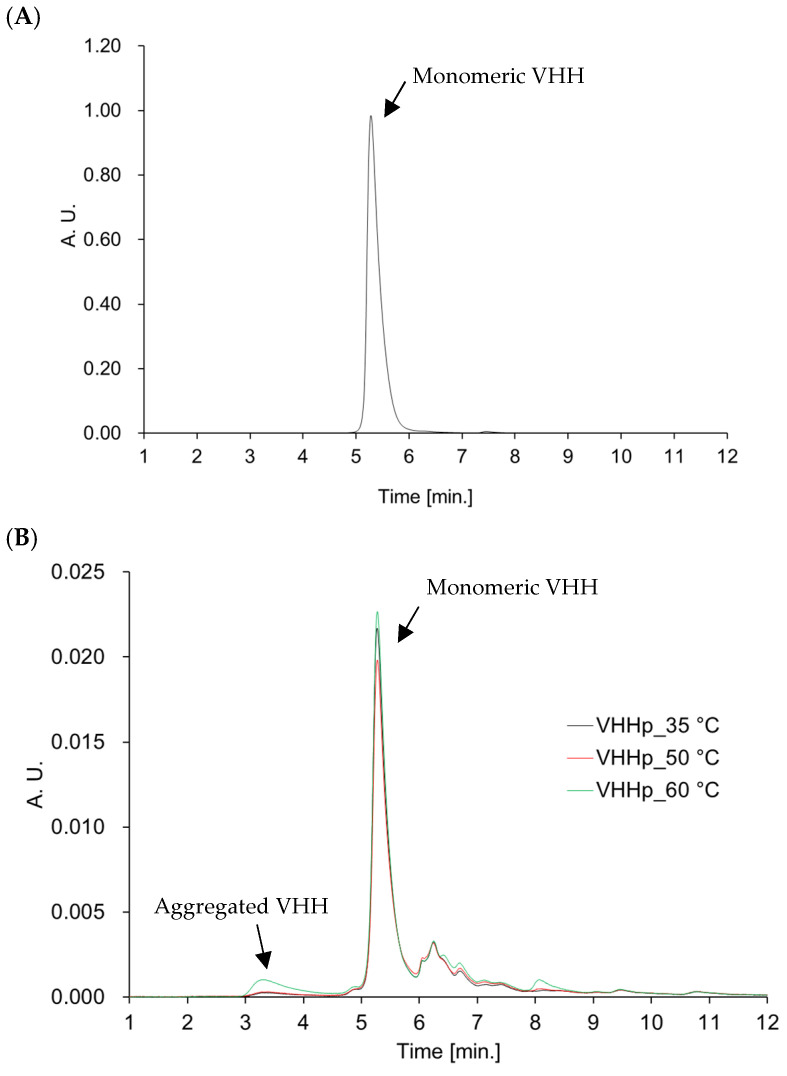
Chromatograms of VHHps. (**A**) Native VHH, (**B**) VHHps prepared at 35 °C, 50 °C, and 65 °C. The vertical axis shows absorbance at 280 nm, and the horizontal axis shows elution time. VHH monomers are eluted at around 5–6 min, and aggregated VHHs are eluted at around 3–4 min. Absorption due to degradation of products was observed at 6–9 min. The elution time of excipient was confirmed in Supplementary Information Figure S2.

**Figure 4:**
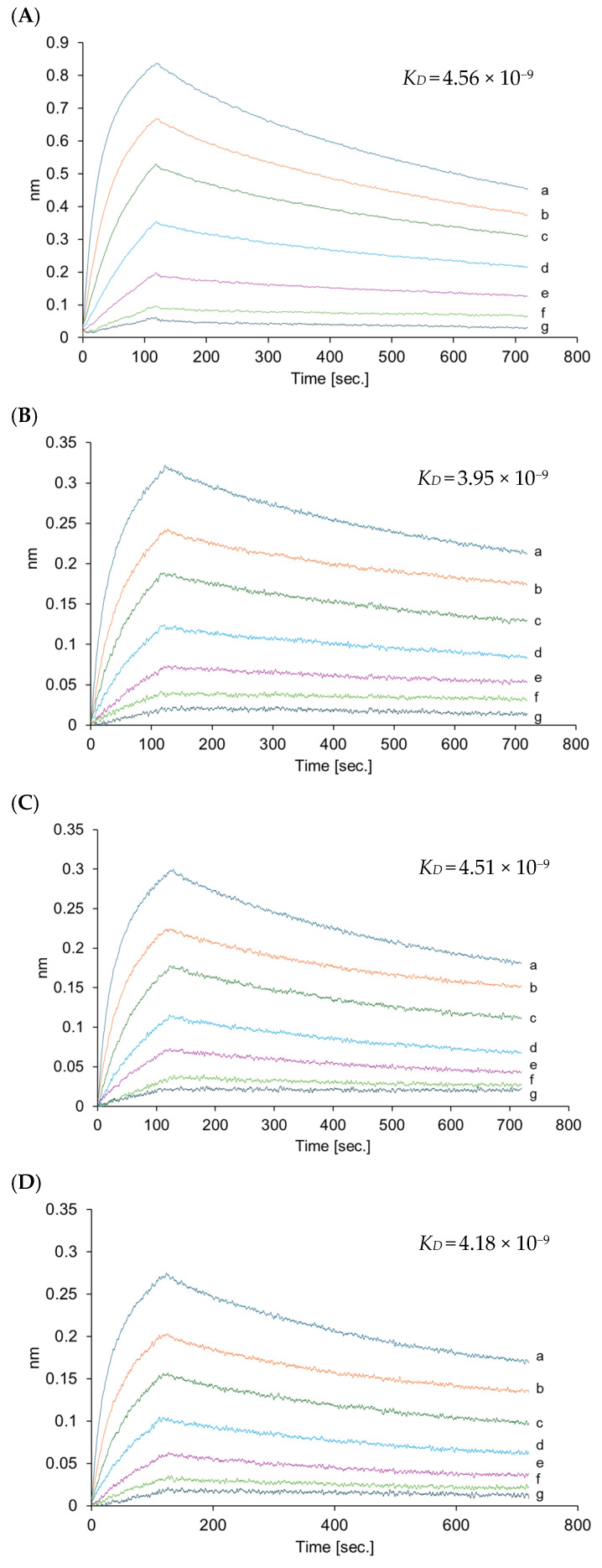
The bio-layer interferometry results for the VHHs. Each graph shows the binding kinetics of VHHs. (**A**) Native VHH, (**B**) VHHp (35 °C), (**C**) VHHp (50 °C), and (**D**) VHHp (65 °C). (a–g) of each graph indicates the binding capacities at different VHH concentrations: (a) 200, (b) 100, (c) 50, (d) 25, (e) 12.5, (f) 6.2, and (g) 3.1 nmol/L in the bio-layer interferometry analysis.

The VHH (MW; 14667.1 Da, *T*_m_; 64.9 °C, *T*_agg_; 48.8 °C) in VHHps may be affected by environmental conditions during the FDD process. Therefore, HPLC analysis and bio-layer interferometry were conducted to clarify whether the physicochemical characteristics of the VHH were altered after the FDD process (Table 1). HPLC analysis confirmed that the VHH aggregated in VHHps (Figure 3 and Figure S2). Regardless of the number of aggregates, the binding activity of the VHH in each VHHp did not change when compared with VHH before powderization (Figure 4). A previous study reported that the temperature during the manufacturing process is a critical factor responsible for aggregation [17]. The aggregation ratio of the VHH in VHHps was dependent on the airflow temperature, with the highest aggregation ratio of 9% observed under the airflow conditions of 65 °C. The correlation between the aggregation ratio and airflow temperature can be partly explained by the aggregation temperature (*T*_agg_) of the VHH. The *T*_agg_ of the VHH was determined to be 48.8 ± 8.4 °C, which is similar to the temperature of the air drying during the FDD process. This observation may explain why the observed aggregation ratio under the airflow conditions of 65 °C was clearly high compared with other VHHps. In this context, the structure of the VHH was influenced by the environment of the powderization process, and mild conditions may be required for VHHp powderization. Nonetheless, the aggregation ratios of the VHH for every VHHp sample were below 10%. This observation may explain the similar binding affinities of each VHHp in the bio-layer interferometry analysis. On the other hand, since the wavelength shift (nm) of the interference wave is smaller than that of the native VHHp (Figure 4A), no aggregation occurs regardless of the temperature, but decomposition or inactivation occurs. The maximum nm value for 200 nM HSA captured on the immobilized VHH is higher for the native material (0.85 nm) in comparison with the VHHp samples (0.3 nm): around 0.85 nm for the original and around 0.3 nm for powdered VHH samples with 200 nM HSA. This indicates that only 30% of the VHH of the powdered sample has been functionally immobilized on the sensor surface. Thus, with this powderization method, it is recommended to use approximately three times the amount of VHH desired. In addition, the effect of the long-term storage of VHHps on VHH function is another important issue. Especially, the ability to store antibodies at room temperature offers great opportunities for transporting antibodies without refrigeration, especially to Africa and Southeast and South Asia. We measured the affinity of VHHs in each VHHp stored at room temperature for over two years (Figure S3). We found that there was almost no change in the affinity. However, bio-layer interferometry showed that about 10% degradation or inactivation had occurred. Although some degradation was observed, the fact that there was no change in the VHH function after storage at room temperature for over a year indicates the stability of VHHs and the usefulness of powderization. These insights may facilitate various applications of VHHs for pharmaceutical use, especially for preparing dry powder inhalers. By designing the function of VHHs through evolutionary engineering [24] or designing multivalent VHHs through protein engineering [25], inhalable VHH drugs may potentially be utilized for treating lung cancer, chronic obstructive pulmonary disease, idiopathic interstitial pneumonias, and idiopathic pulmonary fibrosis. Additionally, formulating VHHs with larger particle sizes for neurogenic diseases potentially offers intranasal medications, with peptide drugs reported as promising candidates for treating central nervous system diseases [26]. Inhalation via pulmonary administration may effectively treat systemic diseases with reduced side effects [27], indicating that powdered VHHs produced using FDD technology can be applied to various unmet medical needs.

### 3.3. Inhalation Properties of VHHps

**Figure 5:**
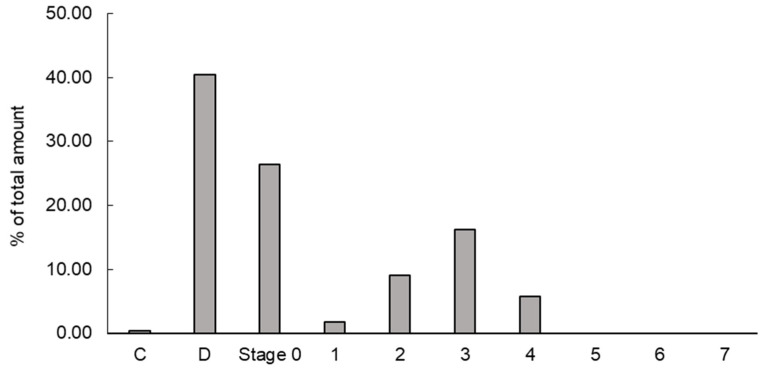
Deposition patterns of VHHps in the cascade impactor (C, capsule; D, device). The cascade impactor installs stages 0–8. Deposition ratios of VHHps were calculated by the deposition amounts of VHHps at each stage.

The VHHp generated via airflow drying at 35 °C was used to evaluate the inhalation properties. The SEM image of the VHHp showed that it had a smooth surface and spherical shape, possibly leading to the good flow ability of this VHHp because of its low surface area-to-volume ratio. To estimate the in vivo deposition of the VHHp after inhalation, the in vitro inhalation properties were evaluated via Andersen cascade impactor analysis. The deposition pattern of the VHHp after inhalation with JetHelar^®^, a simple inhalation device, is shown in Figure 5. The calculated FPF value of this VHHp was 31.1%, representing a fine inhalation property. The FPF value of mannitol particles with a diameter of 4 mm was previously evaluated, and the FPF was reported to be 28.3% at an airflow of 60 L/min [28]. The airflow of the past study was approximately two-fold higher than that used for this VHHp. In this context, the VHHp may have finer inhalation properties than the particles produced by the general spray-drying method. However, the FPF value generally tends to vary depending on the measurement conditions and device. The findings indicate the applicability of this VHHp as an inhalable powder for intratracheal administration. Generally, the powder produced via crushing after freeze-drying is not only non-uniform in size, but often has a diameter exceeding 100 μm. In addition, the spray-drying method makes it easy to produce spherical powder, but the particle size is approximately 10 μm to 500 μm, making it difficult to produce the 5 μm particles required in this research. Conventional particles with large diameters, especially when used as pharmaceuticals, are likely to fail to reach the alveoli and cause thrombosis. In addition, a tendency for deposition at tracheal bifurcations has been observed, and it is feared that this may lead to interstitial pneumonia.

## 4. Conclusions

The micro-sized VHHp prepared using the FDD process had a spherical shape and smooth surface with a narrow size distribution. The amount of aggregated VHH in the VHHp depended on the airflow temperature used in the FDD process, and low temperatures provided lower aggregation amounts of the VHH. The binding capabilities of VHHs in the VHHps were very similar in each sample, although the amounts of aggregated VHH in each sample differed. VHHps displayed fine inhalation properties in the cascade impactor test, indicating suitability for pharmaceutical applications, especially as an inhalable formulation. These findings show that the FDD process may be suitable for preparing various VHH powder formulations, including pharmaceutical formulations.

## Figures and Tables

**Table 1:** HPLC and bio-layer interferometry analysis of prepared VHHps.

	Native VHH	VHHp (35 °C)	VHHp (50 °C)	VHHp (65 °C)
Aggregation (%)	0.00	1.77	3.09	9.06
*K*_D_ (M)	4.56 × 10^−9^	3.95 × 10^−9^	4.51 × 10^−9^	4.18 × 10^−9^
*k*_on_ (M/s)	2.08 × 10^5^	1.57 × 10^5^	1.74 × 10^5^	1.86 × 10^5^
*k*_off_ (s^−1^)	9.48 × 10^−4^	6.18 × 10^−4^	7.87 × 10^−4^	7.75 × 10^−4^

## Data Availability

The original contributions presented in this study are included in the article/Supplementary Material. Further inquiries can be directed to the corresponding authors.

## Supplementary Materials

The following supporting information can be downloaded at: https://www.mdpi.com/article/10.3390/antib14020029/s1, Figure S1. Schematic diagram of the cascade impactor analysis system. Figure S2. Chromatogram of mannitol. The vertical axis shows absorbance at 280 nm, and the horizontal axis shows elution time. Mannitol has an elution peak at about 6 min. The elution time of mannitol is same as one of the excipients in Figure 3. Figure S3. The bio-layer interferometry analysis of VHHs stored long term as VHHp. VHHs were stored as VHHp in a desiccator at room temperature for 2 years and 4 months. The VHHs in (A–C) are the same as those in (B–D) of Figure 4, respectively.
