# Supplementary material for: A Novel Method for Preparing Uniform Micro-Sized Dry Powder Formulations, Including Aggregation-Controlled VHH
Source: Antibodies (Basel). 2025 Mar 31;14(2):29. doi: 10.3390/antib14020029 (PMC12015861; doi:10.3390/antib14020029)
Supplement: Supplementary file 1 [file antibodies-14-00029-s001.zip › antibodies-3267014-supplementary.pdf]

Supplementary Materials

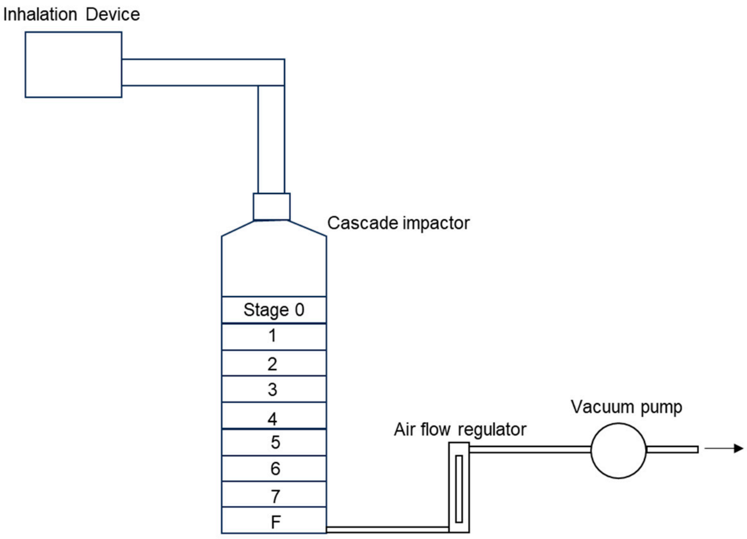

Figure S1. Schematic diagram of the cascade impactor analysis system.

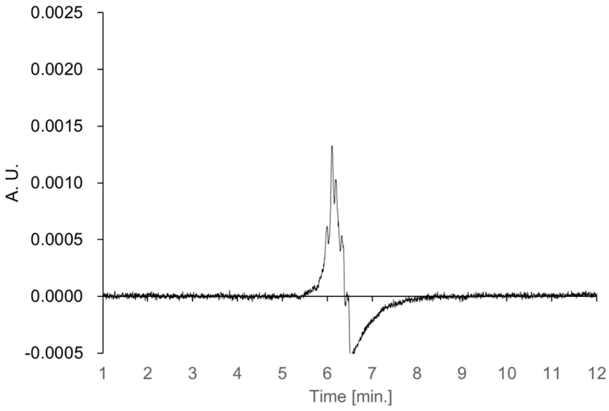

Figure S2. Chromatogram of mannitol. The vertical axis shows absorbance at 280 nm, and the horizontal axis shows elution time. Mannitol has an elution peak at about 6 minutes. The elution time of mannitol is same as one of the excipients in Figure 3.

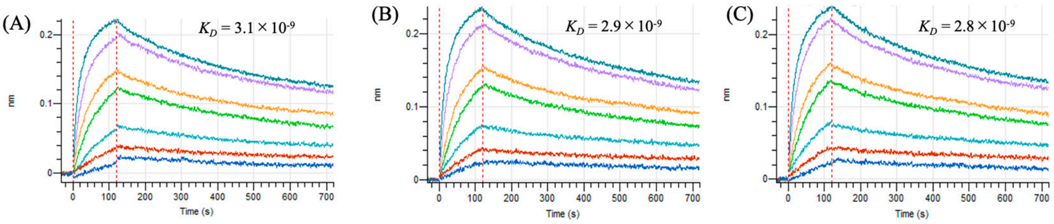

Figure S3. The bio-layer interferometry analysis of VHHs stored long term as VHHp. VHHs were stored as VHHp in a desiccator at room temperature for 2 years and 4 months. The VHHs in (A), (B), and (C) are the same as those in (B), (C), and (D) of Figure 4, respectively.
